# Cilostazol inhibits hyperglucose-induced vascular smooth muscle cell dysfunction by modulating the RAGE/ERK/NF-κB signaling pathways

**DOI:** 10.1186/s12929-019-0550-9

**Published:** 2019-09-06

**Authors:** Sheng-Chiang Su, Yi-Jen Hung, Chia-Luen Huang, Yi-Shing Shieh, Chu-Yen Chien, Chi-Fu Chiang, Jhih-Syuan Liu, Chieh-Hua Lu, Chang-Hsun Hsieh, Chien-Ming Lin, Chien-Hsing Lee

**Affiliations:** 1 Division of Endocrinology and Metabolism, Department of Internal Medicine, Tri-Service General Hospital, National Defense Medical Center, Taipei, Taiwan; 2 School of Dentistry, National Defense Medical Center, Taipei, Taiwan; 3 Department of Oral Diagnosis and Pathology, Tri-Service General Hospital, National Defense Medical Center, Taipei, Taiwan; 4 Graduate Institute of Medical Sciences, National Defense Medical Center, Taipei, Taiwan; 5 Department of Pediatrics, Tri-Service General Hospital, National Defense Medical Center, Taipei, Taiwan; 6 Division of Biochemistry, National Defense Medical Center, Taipei, Taiwan

**Keywords:** Cilostazol, Vascular smooth muscle, RAGE, Diabetes

## Abstract

**Background:**

Increasing evidence suggests that high glucose (HG) causes abnormalities in endothelial and vascular smooth muscle cell function (VSMC) and contributes to atherosclerosis. Receptor for advanced glycation end-products (RAGE) has been linked to the pathogenesis of both the macrovascular and microvascular complications of diabetes. Cilostazol is used to treat diabetic vasculopathy by ameliorating HG-induced vascular dysfunction.

**Objectives:**

In this study, we investigated whether the cilostazol suppression of HG-induced VSMC dysfunction is through RAGE signaling and its possible regulation mechanism.

**Method:**

We investigated the effect of HG and cilostazol on RAGE signaling in A7r5 rat VSMCs. Aortic tissues of *streptozotocin* (STZ) diabetic mice were also collected.

**Results:**

Aortic tissue samples from the diabetic mice exhibited a significantly decreased RAGE expression after cilostazol treatment. HG increased RAGE, focal adhesion kinase (FAK), matrix metalloproteinase-2 (MMP-2), intercellular cell adhesion molecule-1 (ICAM-1) and vascular cell adhesion molecule-1 (VCAM-1) expressions, and was accompanied with increased reactive oxygen species (ROS), cell proliferation, adhesion and migration. Cilostazol significantly reversed HG-induced RAGE, ROS, downstream gene expressions and cell functions. RAGE knockdown significantly reversed the expressions of HG-induced vasculopathy related gene expressions and cell functions. Cilostazol with RAGE knockdown had additive effects on downstream ERK/NF-κB signaling pathways, gene expressions and cell functions of A7r5 rat VSMCs in HG culture.

**Conclusions:**

Both in vitro and in vivo experimental diabetes models showed novel signal transduction of cilostazol-mediated protection against HG-related VSMC dysfunction, and highlighted the involvement of RAGE signaling and downstream pathways.

## Introduction

Type 2 diabetes is the most prevalent and serious metabolic disease worldwide [1]. Epidemiological studies have identified diabetes to be an independent risk factor for atherosclerosis-associated morbidity and mortality [2]. In addition, recent studies have demonstrated that chronic hyperglycemia and reactive oxygen species (ROS) are involved in the development of atherosclerosis through various pathways. Moreover, ROS have been shown to be involved in the progression of endothelial cell dysfunction, proliferation and migration of vascular smooth muscle cells (VSMCs), and expressions of adhesion molecules such as intercellular adhesion molecule-1 (ICAM-1) and vascular cell adhesion molecule-1 (VCAM-1) [3].

The receptor for advanced glycation end-products (RAGE) was initially identified as the signal transducing-receptor of advanced glycation end-products (AGEs) [4]. Driven by sustained hyperglycemia and oxidative stress, enhanced AGE generation coupled with RAGE hyperactivity has been demonstrated to be a critical pathway involved in diabetic micro and macrovascular complications [5]. RAGE is physiologically expressed in a number of cells involved in immune/ inflammatory responses, including monocytes/macrophages, granulocytes, endothelial cells, VSMCs, and adipocytes [5, 6]. RAGE is a multi-ligand receptor of the immunoglobulin superfamily involved in diverse ligands related to the pathogenesis of atherosclerosis. The exposure of vascular endothelial cells to various RAGE ligands, including AGEs, S100/calgranulins, and high-mobility group box 1 protein (HMGB-1), has been shown to augment RAGE activation resulting in enhanced generation of ROS and activation of the transcription factor NF-κB [7]. In turn, this has been confirmed to lead to sustained upregulation of proinflammatory mediators, adhesion molecules and to the initiation of atherosclerosis [8].

Cilostazol, a phosphodiesterase type 3 (PDE3) inhibitor, has been regarded as possessing antiplatelet and vasodilatory effects, and inducing increased concentrations of intracellular 3′-5′ cyclic adenosine monophosphate (cAMP) levels [9]. Cilostazol acts as a vasodilator, antithrombotic antiplatelet agent, and has been demonstrated to be involved in various stages of the atherosclerotic process [10]. The anti-atherogenic effect of cilostazol has been ascribed to its ability to suppress superoxide, resulting in attenuation of NF-κB activation, VCAM-1/monocyte chemotactic protein-1 (MCP-1) expressions and monocyte recruitment in low-density lipoprotein (LDL) receptor-null mice [11]. In addition, cilostazol has been shown to inhibit VSMC proliferation, thereby improving peripheral blood flow and insulin sensitivity via attenuation of inflammation processes [12]. Recent clinical studies have revealed that cilostazol can have effects of reduced triglyceride levels and increased high-density lipoprotein (HDL) on patients with peripheral arterial occlusive disease (PAOD), thereby improving postprandial lipemia in patients with diabetes [13].

In our previous report, we discovered that cilostazol therapy effectively ameliorated the severity of PAOD, which was defined as an improvement in ankle-brachial index (ABI) in patients with type 2 diabetes. Furthermore, augmentation of the plasma circulating soluble form of RAGE (sRAGE) and attenuation of proinflammatory markers as well as adhesion molecules after cilostazol treatment were also observed. There was also a significant association between the improvement in ABI and the augmentation of plasma sRAGE, and hence enhancement of plasma sRAGE level was regarded as an independent factor of improving the severity of peripheral arterial insufficiency in type 2 diabetic patients [13]. However, it is still unclear whether cilostazol can improve diabetes-associated atherosclerosis through RAGE pathways to influence downstream signaling such as inflammation and adhesion molecules. Therefore, the aim of this study was to investigate the molecular mechanism of cilostazol on the development of diabetic vasculopathy, and whether that was relevant to RAGE and its downstream signaling.

## Materials and methods

### Animal experiments

Male BALB/c mice were obtained from the National Laboratory Animal Breeding and Research Center (Taipei, Taiwan). To induce type 2 diabetes mellitus, the mice were housed in laboratory cages and fed with a high-fat (HF) diet (40% fat, Research Diets, Inc., NJ, USA) [14] for 3 weeks. Subsequently, the mice received 75 mg/kg and 150 mg/kg of intravenous STZ, 5 days apart. Feed was not withheld from any of the animals at the time of STZ administration. After induction, blood glucose was measured daily by tail-vein sampling using a ACCUCHEK glucometer (Roche, Basel, Switzerland). Animals with a blood glucose level more than 11.1 mmol/L (200 mg/dL) were included in this study. Six mice were treated with cilostazol for 2 months, where cilostazol was diluted from 0.5% CMC (carboxymethyl cellulose sodium salt), which was used as a vehicle control. Another six mice were treated with 0.5% CMC for 2 months. Finally, the animals were sacrificed and blood and ascending aorta samples were taken. The Animal Ethics Board of National Defense Medical Center (Taipei, Taiwan) approved all animal experimental procedures.

### Immunohistochemistry assay

Animal tissue samples in paraffin blocks were cut into 4-μm sections, which were dewaxed and subjected to microwave antigen retrieval. Endogenous peroxidase activity and nonspecific binding were blocked by incubation with 3% hydrogen peroxide and non-immune serum, respectively. Slides were then incubated with anti-RAGE antibody (Millipore, #051050, MA, USA) at 1:250 at 4 °C overnight, and anti-rabbit secondary antibody for 1 h. Diaminobenzidine hydrochloride (Dako, Carpinteria, CA, USA) was then added to localize positive staining sequentially by light microscopy. The sections were counterstained with hematoxylin and cover slipped.

### Protein isolation from frozen/OCT-embedded samples

The tissue sections were moved to a 2-ml tube containing ceramic beads (2.8 mm, Bertin Technologies, France). Lysis buffer (50 mM Tris–HCl [pH 7.4], 150 mM NaCl, 2 mM EDTA, 1% NP-40, 0.1% SDS, protease inhibitor) was added, and the samples were homogenized by vortex and grinding in a homogenizer (Precellys®24, Bertin Technologies). The samples were kept on ice for 30 min to complete the lysis reaction followed by centrifugation (4 °C, 13000 g, 15 min) to collect the supernatant. The samples were then collected and stored at − 80 °C.

### Cell culture and reagents

Smooth muscle cell lines from rat thoracic aorta, A7r5 (RRID:CVCL_0137), were purchased from the Bioresource Collection and Research Center (BCRC, Taiwan). Cells were cultured in Dulbecco’s Modified Eagle’s Medium (DMEM) and supplemented with 10% fetal bovine serum (FBS), and incubated at 37 °C in 5% CO2. The cells were seeded at a density of 1.5 × 10^6^ cells on a 10 cm2 dish in DMEM with 10% FBS for 24 h. After serum starvation for 24 h in DMEM with 0.5% FBS, the cells were stimulated by high glucose (HG). A7r5 cells were grown in 30 mmol/L glucose, and controls received 25 mmol/L mannitol and 5 mmol/L glucose. Cilostazol (kindly provided by Otsuka Pharmaceutical Co. Ltd., Tokushima, Japan) was dissolved in dimethyl sulfoxide. The final concentration of dimethyl sulfoxide in the culture medium was less than 0.1%, which had no effect on VSMCs. The cells were incubated for 24 h with 100 and 200 μM cilostazol after high glucose stimulation. All of the chemical compounds, N-acetylcysteine (NAC), U0126 and PDTA were purchased from Cell Signaling (Danvers, CO, USA).

### siRNA transfection

Knockdown of RAGE was performed using specific single or pooled siRNAs purchased from Dharmacon RNAi Technologies (Thermo, MA, USA). SiGENOME non-target siRNAs served as negative controls, and transfection was carried out according to the manufacturer’s protocol.

### Intracellular ROS

A7r5 cells were plated in a 6-well plate, grown to confluence, and harvested by trypsinization. Cell pellets were washed with PBS and centrifuged for 5 min at 1000×g at room temperature. The cell pellets were resuspended in 10 μmol/L of CM-H2DCFDA by gently pipetting up and down. The cells were then incubated in a cell incubator in the dark for 45 min, followed immediately by flow cytometry analysis (BD Bioscience).

### Cell proliferation assay

A7r5 cells were cultured at a density of 1.5 × 10^5^ cells/well in a 24-well plate. The cells were exposed to various stimuli. A methylene blue dye assay was used to evaluate the effect of high glucose and cilostazol on cell growth. The resulting cell growth was measured at 540 nm and calculated graphically in comparison with the growth of the controls.

### Cell adhesion assay

A7r5 cells (1 × 10^6^ cells/ml) were cultured in normoglycemic (5 mmol/L glucose) and hyperglycemic (30 mmol/L glucose) conditions in a 6-well culture plate. The cells were Incubated for 24 h in a CO2 incubator. THP-1 was fluorescence-labeled with calcein-AM by incubating the cells (1 × 10^7^ cells/ml) with 5 μ calcein-AM in RPMI 1640 for 30 min at 37 °C in a CO2 incubator. The cells were washed three times with PBS to remove excess dye and resuspended in phenol red-free RPMI 1640 (with 10% FBS) at a density of 1 × 10^6^ cells/ml. After high glucose and cilostazol treatment, the A7r5 cells were co-cultured with calcein-AM-labeled cells (1 × 10^6^ cells/ml in 6- well) in a CO2 incubator at 37 °C for 1 h. The A7r5 cells were then washed four times with PBS to remove the non-adherent calcein-AM-labeled cells and replaced with 1.0 ml of PBS. The fluorescence of each well was measured using a fluorescence microscopy with excitation and emission wavelengths of 480 nm and 530 nm, respectively.

### Migration assay

The migration ability of A7r5 cells was examined using 24-well culture insert-based assays (BD Biosciences, Franklin Lakes, NJ, USA). The culture insertion, with a pore size of 8 μm, was pre-coated to a density of 100 μg/insert of gelatin (Sigma, MO, USA). Cells were suspended in medium containing 10% NuSerum (Corning, New York, U.S), and 2.5 × 10^4^ cells were added to the insert. After incubating for 10 h at 37 °C, the cells that migrated through a Fluoro-Blok membrane (Corning, New York, U.S) were stained with propidium iodine, and fluorescence images were taken. The cells were then counted with Image J software.

### Western blot analysis

Whole cell lysates for Western blotting were harvested in RIPA buffer (1% SDS and 10 mM Tris buffer, pH 7.4) containing protease and phosphatase inhibitors (Thermo, Wilmington, DE, USA). Protein concentrations in the supernatants were determined using a Pierce BCA Protein Assay Kit (Thermo, Rockford, IL, USA). Thirty micrograms of protein were separated on 5–15% gradient SDS-PAGE gel and transferred to polyvinylidene difluoride membranes (Millipore, Bedford, MA, USA) by wet blotting using an electroblotter (Hoefer system). Membranes were blocked for 1 h at 25 °C with 2% bovine serum albumin or 5% skimmed milk in Tris-buffered saline and Tween 20 (TBST). The membranes were incubated with appropriate dilutions of the primary antibodies: RAGE antibody (SC-74473 [1:1000 dilution]; Santa Cruz, Dallas, USA), I-CAM antibody (SC-8439 [1:1000 dilution]; Santa Cruz, Dallas, USA), V-CAM antibody (SC-13160 [1:1000 dilution]; Santa Cruz, Dallas, USA), FAK antibody (3285 [1:1000 dilution]; Cell Signaling, Danvers, MA, USA), p65 antibody (3033 [1:1000 dilution]; Cell Signaling, Danvers, MA, USA), IκBα antibody (9242 [1:1000 dilution]; Cell Signaling, Danvers, MA, USA), GAPDH antibody (5174 [1:2000 dilution]; Cell Signaling, Danvers, MA, USA), Phospho-JNK antibody (9255 [1:1000 dilution]; Cell Signaling, Danvers, MA, USA), JNK antibody (9252 [1:1000 dilution]; Cell Signaling, Danvers, MA, USA), Phospho-ERK antibody (9101 [1:1000 dilution]; Cell Signaling, Danvers, MA, USA), ERK antibody (9102 [1:1000 dilution]; Cell Signaling, Danvers, MA, USA), MMP2 antibody (13,405 [1:1000 dilution]; Millipore, Danvers, MA, USA), β-actin antibody (600–501 [1:3000 dilution]; Novus, CO, USA), overnight at 4 °C. After being washed in TBST three times, the membranes were incubated for 60 min with HRP-conjugated goat anti-rabbit or anti-mouse secondary antibodies at 25 °C. Signals were visualized using horseradish peroxidase-conjugated secondary antibodies and an enhanced chemiluminescence assay. Band intensities were determined using a UVP (ChemStudio series imagers) imaging system.

### Statistical analysis

Values are expressed as means ± SEM. Immunoblot data are expressed as means ± SEM of band intensity relative to the controls. All experiments were repeated for three times. Groups were analyzed for differences by one-way ANOVA followed by Tukey’s test. Significance was considered at *P* < 0.05. All statistical analyses were performed using SPSS software (version 20.0; Chicago, II, USA).

## Results

### Effects of cilostazol on the artery expression of RAGE and possible associated signaling pathways in the mice with STZ-induced diabetes

**Fig. 1:**
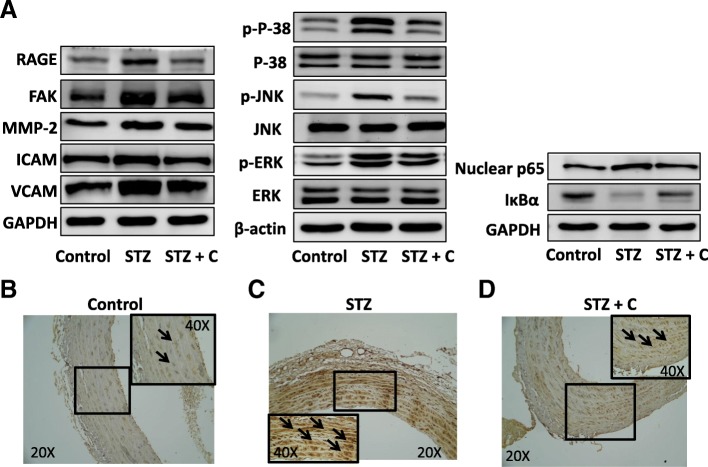
RAGE and possible associated signaling pathways were elevated in STZ-induced diabetic mice and attenuated after cilostazol treatment. **a**, protein levels of RAGE and possible associated signaling molecules in vascular tissues in mice treated with STZ and/or cilostazol were measured by Western blot analysis. Immunohistochemical staining of RAGE was analyzed in the intima layer of vessels in mice from the control group (**b**), STZ-induced diabetes (**c**) and combined treatment with STZ and cilostazol (**d**). Western blots were independently repeated three to six times GAPDH and β-actin served as loading control

In order to investigate the role of RAGE and possible associated signaling pathways in diabetic vasculopathy, the expression of RAGE and possible downstream signaling molecules were determined by western blot in the aortas of the STZ-induced diabetic mice. The protein expression of RAGE, focal adhesion kinase (FAK), matrix metalloproteinase-2 (MMP-2), intercellular cell adhesion molecule-1 (ICAM-1) and vascular cell adhesion molecule-1 (VCAM-1) were higher in the STZ diabetic mice compared to the control mice, and the expression of RAGE, FAK, MMP-2, ICAM-1 and VCAM-1 were significantly decreased after cilostazol treatment (Fig. 1a). Consistent with the above molecules expression levels, the STZ diabetic mice exhibited both an increased protein expression of RAGE and downstream signaling pathways (P-38, JNK, ERK phosphorylation and nuclear p65 expression and downregulated the expression of IκBα) compared to the control group, and significantly decreased RAGE and downstream signaling proteins expression after cilostazol treatment (Fig. 1a). We further evaluated the distribution and expression of RAGE in the aortas by immunohistochemical staining, and found that RAGE was mainly detected in both the endothelium and VSMCs. In addition, the staining of RAGE protein was more prominent in the aortic sections of the STZ diabetic mice compared to the control mice. After cilostazol treatment, the staining of RAGE protein was decreased in the STZ diabetic mice (Fig. 1b, c and d).

### Effects of HG concentration and cilostazol on the protein expressions of RAGE, FAK, MMP-2, ICAM-1 and VCAM-1

**Fig. 2:**
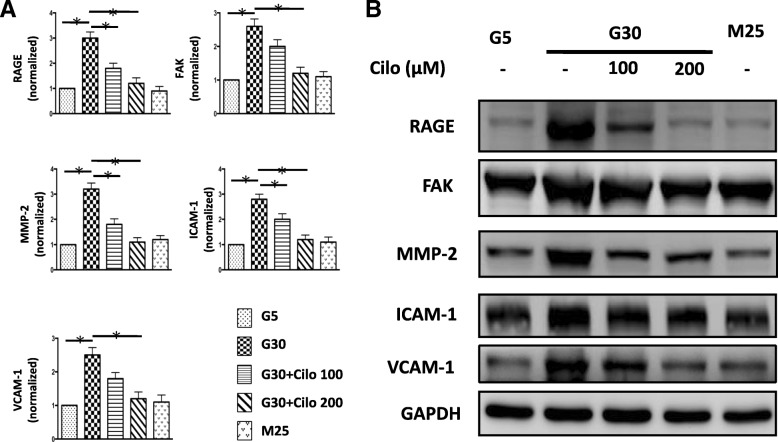
The expressions of RAGE and molecules involved in adhesion, migration and inflammation were significantly increased under hyperglycemic condition and were diminished in response to cilostazol treatment in a dose-dependent manner. **a**, densitometric quantification of Western blot bands (*n* = 3 /group). **b**, protein expressions of RAGE, MMP-2, VCAM-1, ICAM-1 and FAK were examined by Western blot analysis. A7r5 cells were incubated for 24 h with 100 and 200 μM cilostazol after stimulation with 5 and 30 mM glucose. Mannitol was used as an osmolality control. Western blots were independently repeated three times, and the representative data are shown. GAPDH served as a loading control. *, statistically significant differences, *p* < 0.05 by the Student’s t test. Error bars, SEM of three independent experiments

To determine whether HG and cilostazol affects the expression of RAGE in VSMCs, A7r5 cells were incubated for 24 h with 100 and 200 μM cilostazol after stimulation with 5 and 30 mM glucose. After 30 mM glucose treatment, the protein expressions of RAGE, FAK, MMP-2, ICAM-1 and VCAM-1 were significantly increased (Fig. 2a and b). We then investigated the effect of cilostazol on HG-induced VSMC dysfunction, and the results showed a significant suppression of RAGE, FAK, MMP-2, ICAM-1 and VCAM-1 expressions in a dose-dependent manner (Fig. 2a and b).

### Effects of cilostazol on HG-induced oxidative stress, proliferation, adhesion and migration in A7r5 cells

**Fig. 3:**
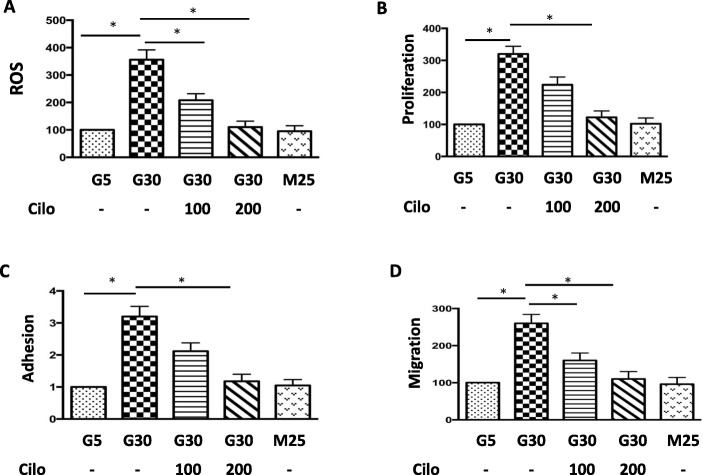
Effects of HG concentration and cilostazol on A7r5 cell ROS expression, proliferation, adhesion, and migration. **a**, **b**, **c**, **d** Representative of HG concentration and cilostazol on A7r5 cell ROS expression, proliferation, adhesion, and migration. The expressions and functions were increased by HG and decreased after cilostazol treatment. Mannitol was used as an osmolality control. *, statistically significant differences, p < 0.05 by the Student’s t test. Error bars, SEM of three independent experiments

To further evaluate HG-induced oxidative stress and associated VSMC dysfunction, we examined ROS production and cell proliferation, adhesion and migration of VSMCs. After 30 mM glucose treatment, the ROS production and cell proliferation, adhesion and migration of VSMCs were significantly increased (Fig. 3a-d). We then investigated the effect of cilostazol on HG-induced VSMC dysfunction, and the results showed a significant dose-dependent decrease in ROS production and reversal of cell proliferation, adhesion and migration after the addition of cilostazol (Fig. 3a-d).

### Effects of cilostazol on HG-induced expression of RAGE and associated signaling pathways in A7r5 cells

**Fig. 4:**
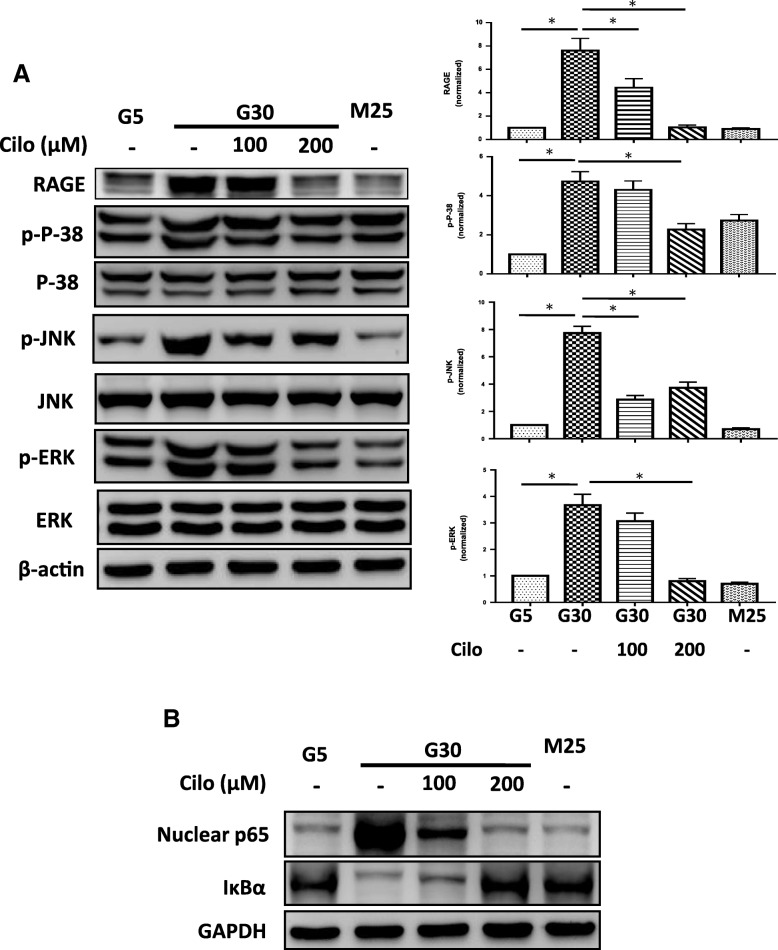
Effects of HG concentration and cilostazol on A7r5 cell downstream signaling pathways. **a** Representative immunoblots of RAGE, p-P38, p-JNK and p-ERK expressions under treatment with HG concentration and cilostazol on A7r5 cells. The expressions were increased by HG and decreased after cilostazol treatment. **b** Representative immunoblots of nuclear p65 and IκBα expressions under treatment with HG concentration and cilostazol on A7r5 cells. Mannitol was used as an osmolality control. Western blots were independently repeated three times, and the representative data are shown. GAPDH served as a loading control. *, statistically significant differences, *p* < 0.05 by the Student’s t test. Error bars, SEM of three independent experiments

Previous studies have reported that RAGE has many diverse signaling capabilities through P-38, JNK, ERK, and NF-κB pathways by which it regulates cell function. In this study, we detected HG-induced A7r5 cell dysfunction including cell proliferation, adhesion and migration. To further elucidate the downstream signaling pathways of RAGE after HG treatment, we examined P-38, JNK, ERK, and NF-κB signaling which are known to be highly associated with proliferation, adhesion and migration functions. Along with an increased expression of RAGE, HG significantly activated P-38, JNK, ERK phosphorylation and nuclear p65 expression and downregulated the expression of IκBα after treatment, and cilostazol significantly suppressed and reversed the effect of HG (Fig. 4a, b).

### Effect of cilostazol and RAGE knockdown on HG-induced RAGE signaling pathways and associated functional proteins in A7r5 cells

**Fig. 5:**
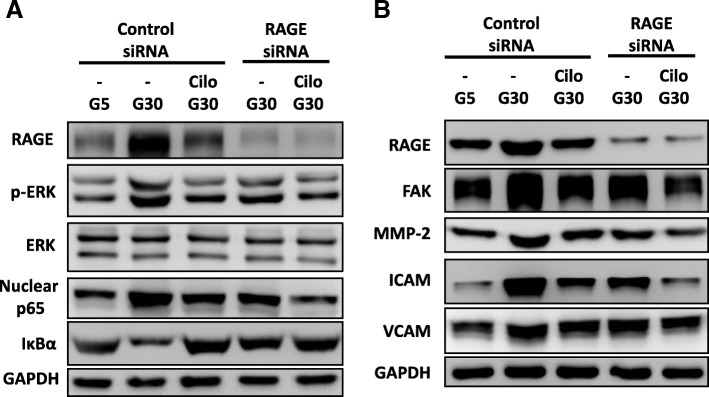
Effects of cilostazol and RAGE knockdown on HG concentration-induced signaling pathways and FAK, MMP-2**,** ICAM-1 and VCAM-1 expressions in A7r5 cells. **a** Representative immunoblots of RAGE, p-ERK, nuclear p65 and IκBα expressions under treatment with HG, cilostazol and RAGE knockdown. **b** Representative immunoblots of RAGE, FAK, MMP-2, ICAM-1 and VCAM-1 expressions under treatment with HG, cilostazol and RAGE knockdown. Western blots were independently repeated three times, and the representative data are shown. GAPDH served as a loading control

In order to confirm the effects of HG treatment and cilostazol on A7r5 cells through RAGE signaling, we investigated whether RAGE knockdown in A7r5 cells had a similar effect to cilostazol on HG culture. The A7r5 cells were infected with RAGE siRNA and control siRNA vectors and cultured in 30 mM glucose. We detected significant changes in the downstream signaling pathways (ERK phosphorylation and nuclear p65 expression and downregulated IκBα expression) and associated functional proteins (FAK, MMP-2, ICAM-1 and VCAM-1 expressions) of the RAGE-siRNA-infected A7r5 cells in HG culture compared with the control-siRNA-infected A7r5 cells in HG culture (Fig. 5a, b). Furthermore, compared with cilostazol or RAGE knockdown alone, we found additive effects in the downstream signaling and associated proteins of the cilostazol and RAGE knockdown A7r5 cells (Fig. 5a, b). These results suggested that RAGE signaling was responsible for cilostazol and HG-induced VSMC dysfunction.

### ROS generation was involved in the HG and cilostazol effect in A7r5 cells

**Fig. 6:**
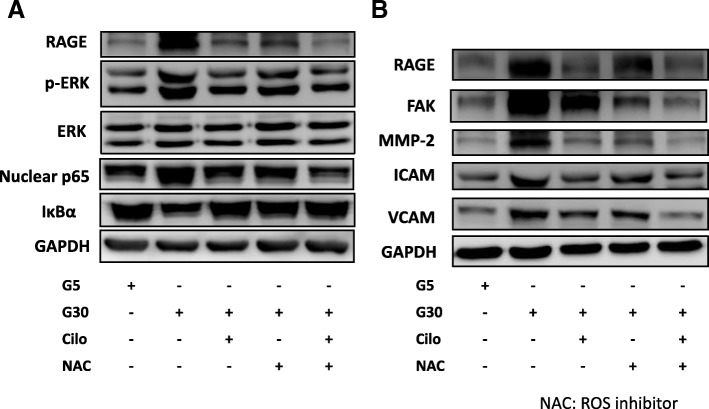
ROS expression was involved in the HG and cilostazol effects on A7r5 cells. **a** Representative immunoblots of RAGE, p-ERK, nuclear p65 and IκBα expressions under treatment with HG, cilostazol and an ROS inhibitor (NAC). **b** Representative immunoblots of RAGE, FAK, MMP-2, ICAM-1 and VCAM-1 expressions under treatment with HG, cilostazol and NAC. Treatment with HG 30 mM and cilostazol 200 μM combined with NAC in A7r5 cells. Western blots were independently repeated three times, and the representative data are shown. GAPDH served as a loading control

ROS are induced under diabetic conditions, and may be involved in the progression of pancreatic β-cell dysfunction, insulin resistance and atherosclerosis found in patients with type 2 diabetes. Clinically, drugs which have anti-oxidant effects may have beneficial effects on the pathogenesis and complications of diabetes. We found that HG induced ROS production, and that this effect was decreased after cilostazol treatment (Fig. 3a). We further used an ROS inhibitor, NAC, to confirm that ROS were involved in HG-induced RAGE signaling pathways and associated functional proteins in the A7r5 cells. The data revealed significant changes in the downstream signaling pathways (ERK phosphorylation and nuclear p65 expression and downregulated IκBα expression) and associated functional proteins (RAGE, FAK, MMP-2, ICAM-1 and VCAM-1 expressions) in HG cultured A7r5 cells compared with NAC-treated A7r5 cells in HG culture (Fig. 6a, b). Compared with cilostazol or NAC alone in HG culture conditions, we found additive effects in the downstream signaling and associated proteins of cilostazol and NAC co-treated A7r5 cells (Fig. 6a, b). These results confirmed that ROS were involved in HG-induced RAGE signaling and VSMC dysfunction, and that these effects were subsequently reversed by cilostazol treatment.

### RAGE-ERK-NF-κB pathways were involved in the HG and cilostazol effect on A7r5 cells

**Fig. 7:**
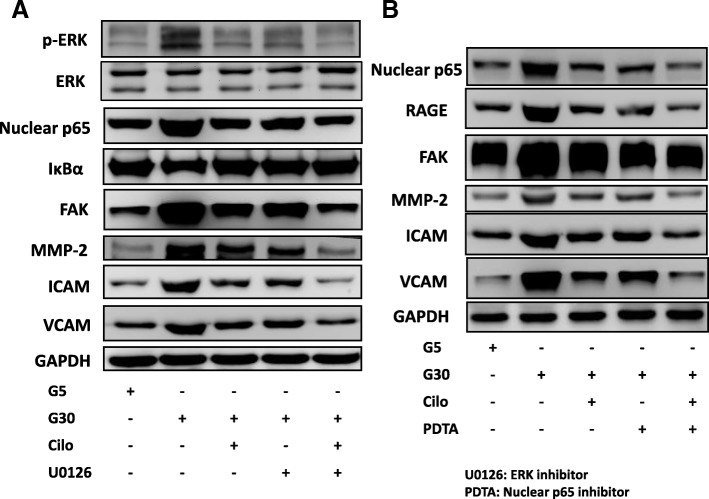
RAGE-ERK-NF-κB was involved in the HG and cilostazol effects on A7r5 cells. **a** Representative immunoblots of RAGE, p-ERK, nuclear p65, IκBα, FAK, MMP-2, ICAM-1 and VCAM-1 expressions under treatment with HG, cilostazol and an ERK inhibitor (U0126). **b** Representative immunoblots of nuclear p65, RAGE, FAK, MMP-2, ICAM-1 and VCAM-1 expressions under treatment with HG, cilostazol and a nuclear p65 inhibitor (PDTA). Treatment with HG 30 mM and cilostazol 200 μM combined with U0126 and PDTA in A7r5 cells. Western blots were independently repeated three times, and the representative data are shown. GAPDH served as a loading control

In order to confirm that the effects of HG and cilostazol on the A7r5 cells were through RAGE-ERK-NF-κB signaling, we added an ERK inhibitor (U0126) and nuclear p65 inhibitor (PDTA) and then cultured the cells in 30 mM glucose. The results showed significant changes in the downstream signaling pathways (ERK phosphorylation and nuclear p65 expression and downregulated IκBα expression) and associated functional proteins (FAK, MMP-2, ICAM-1 and VCAM-1 expressions) in the HG cultured A7r5 cells compared with the U0126-treated A7r5 cells in HG culture (Fig. 7a). Further, compared with cilostazol or U0126 alone in HG culture conditions, we found additive effects in the downstream signaling and associated proteins of cilostazol and U0126 co-treated A7r5 cells (Fig. 7a). Similarly, there were significant changes in the downstream signaling and associated functional proteins after PDTA treatment in the HG cultured A7r5 cells (Fig. 7b).

## Discussion

It has long been appreciated that diabetes contributes to the risk of developing coronary artery disease and multiple studies have demonstrated that hyperglycemia is strongly associated with coronary artery disease pathogenesis [15]. Hyperglycemia not only disrupts protein kinase C signaling and increases oxidative stress, but also enhances formation of AGEs and deteriorates vascular endothelial cell function that result in vascular inflammation, vasoconstriction, thrombosis and atherogenesis [16]. Based on current literatures, both inflammation and oxidative stress are regarded as the main etiology to lead to pathogenesis and progression of atherosclerosis in diabetes [17]. Increasing evidence has shown that the mechanisms of RAGE and its ligand families contribute to the pathogenesis of diabetes and to its complications in vivo and in vitro. In vitro, RAGE ligands have been shown to stimulate the migration and proliferation of VSMCs isolated from mice aorta [18]. In vivo, RAGE ligands were shown to be increased in a diabetic atherosclerotic apoE^−/−^ mouse model compared to nondiabetic apoE^−/−^ controls [19]. In addition, diabetic RAGE^−/−^/apoE^−/−^ double knockout mice have been shown to display decreased leukocyte recruitment, decreased proinflammatory markers, and decreased markers of oxidative stress [20]. Furthermore, significant and strong associations between cardiovascular dysfunction, vascular endothelial damage and enhanced expression of AGE-RAGE axis were observed in the clinical research [21]. Therefore, the possible therapeutic agent with its related molecular mechanism in which RAGE and its downstream signaling are involved specific to development of diabetic vasculopathy is warranted. In current research, our data from in vitro and in vivo studies provided a new mechanism of cilostazol in diabetes-related atherosclerosis. In our in vitro study, significantly decreased RAGE expression in A7r5 cells with cilostazol treatment in a dose-dependent manner was apparently observed. Furthermore, it was firmly confirmed by loss of RAGE expression test that cilostazol improved VSMC functions under HG conditions through RAGE-dependent pathways. Similarly, significant downregulation of RAGE expression was demonstrated in aortic VSMCs in the experimental murine diabetes model after cilostazol treatment.

Cilostazol, a selective PDE-III inhibitor, acts as a vasodilator and antithrombotic antiplatelet agent, and it has been shown to promote lower triglyceride levels and increase HDL in patients with PAOD [22], to improve postprandial lipemia in patients with diabetes [13], to increase nitric oxide (NO) expression with a positive effect on apoptosis [23], to prevent thrombosis after stenting [24], and to have the ability to ameliorate atherosclerotic progression [25]. Cilostazol has also been reported to cause the accumulation of cAMP in VSMCs, resulting in the upregulation of antioncogenes p53 and p21 and hepatocyte growth factor [26]. Increased suppression of the p53 protein in the cellular cycle induces apoptosis in VSMCs, causing an antiproliferative effect. Our previous findings showed that cilostazol inhibited the uremic toxin, P-cresol, induced VSMC proliferation, anti-apoptosis and migration by inhibiting the phosphorylation of PLCγ, Akt and ERK [27]. Furthermore, cilostazol was demonstrated to reduce the levels of soluble adhesion molecules (ICAM-1 and VCAM-1) and expression of inflammatory factors, such as IL-1β, IL-6 and TNF-α in plasma of patients, suggestive of anti-inflammatory and angio-protective roles [28].

However, diabetes related vasculopathy is considered to be complicated rather than vascular disease alone because diabetes has various metabolic effects on macro- and microcirculation in numerous vascular beds [29]. Diabetes is associated with the formation of the ROS by different pathogenic pathways and long-term complications of diabetes mellitus are associated with various oxidative reactions as well as increased free radical generation [30]. Free radicals are involved throughout the atherogenic process beginning from endothelial dysfunction, which is relevant to decline NO levels. Moreover, ROS increase the expressions of various adhesion molecules such as ICAM-1 and VCAM-1, leading to inflammatory cell recruitment. Finally, ROS increase the expressions of various growth factors and activate various stress signaling such as JNK and Pim-1, leading to the proliferation of VSMCs. Hyperglycemia also induces ROS through activation of the glycation reaction and electron transport chain in mitochondria. In addition, AGEs, insulin, and angiotensin II can also induce ROS through activation of NADPH oxidase. ROS are involved in the progression of atherosclerosis which is often observed as a macroangiopathy under diabetic conditions [31]. Platelet and vascular stimulation result in the release of ROS that are known to influence vascular reactivity and thrombosis. Cilostazol may suppress the formation of ROS in platelets and endothelial cells and improve cellular redox status. It may also have a quenching effect on the production of hydroxyl radicals, as well as on oxidative DNA breakage by hydroxyl radicals. Recent studies have suggested that the pleiotropic effects of cilostazol are through an anti-oxidant effect and anti-apoptotic effects in patients and animal models of type 2 diabetes [32]. These effects are mainly through the regulation of inflammatory cytokines such VCAM-1 [33] and ICAM-1 [34]. In a previous study, cilostazol was shown to cause a decrease in the expression of ICAM-1 via NO production in human umbilical vein endothelial cells under hyperglycemic conditions [35]. In an STZ-induced DM nephropathy rat model, cilostazol decreased the activity of ROS and could improve the levels of serum cholesterol, triglycerides, and LDL-cholesterol [36]. In the current study, our data showed that cilostazol inhibited HG-induced ROS production in a dose-dependent manner in VSMCs. Furthermore, we used an ROS inhibitor to clarify that cilostazol may improve VSMC functions under HG conditions through inhibiting ROS activity and RAGE/ERK/NF-κB signaling pathways. Besides, we found increased RAGE protein expressions in the aortic VSMCs of STZ-induced diabetic rats, and that the RAGE expression in aortic tissues was significantly decreased after cilostazol treatment. Furthermore, in vitro, our results first revealed that cilostazol decreased the expressions of RAGE, FAK, MMP-2, VCAM-1 and ICAM-1 in HG cultured A7r5 cells, and also improved the proliferation, adhesion and migration of A7r5 cells. Thus, our present study strongly supported that cilostazol may have anti-oxidative and anti-inflammatory effects, thereby delaying the development of diabetic angiopathy, which was similar to conclusions of the study conducted by Yeh et al. [37].

**Fig. 8:**
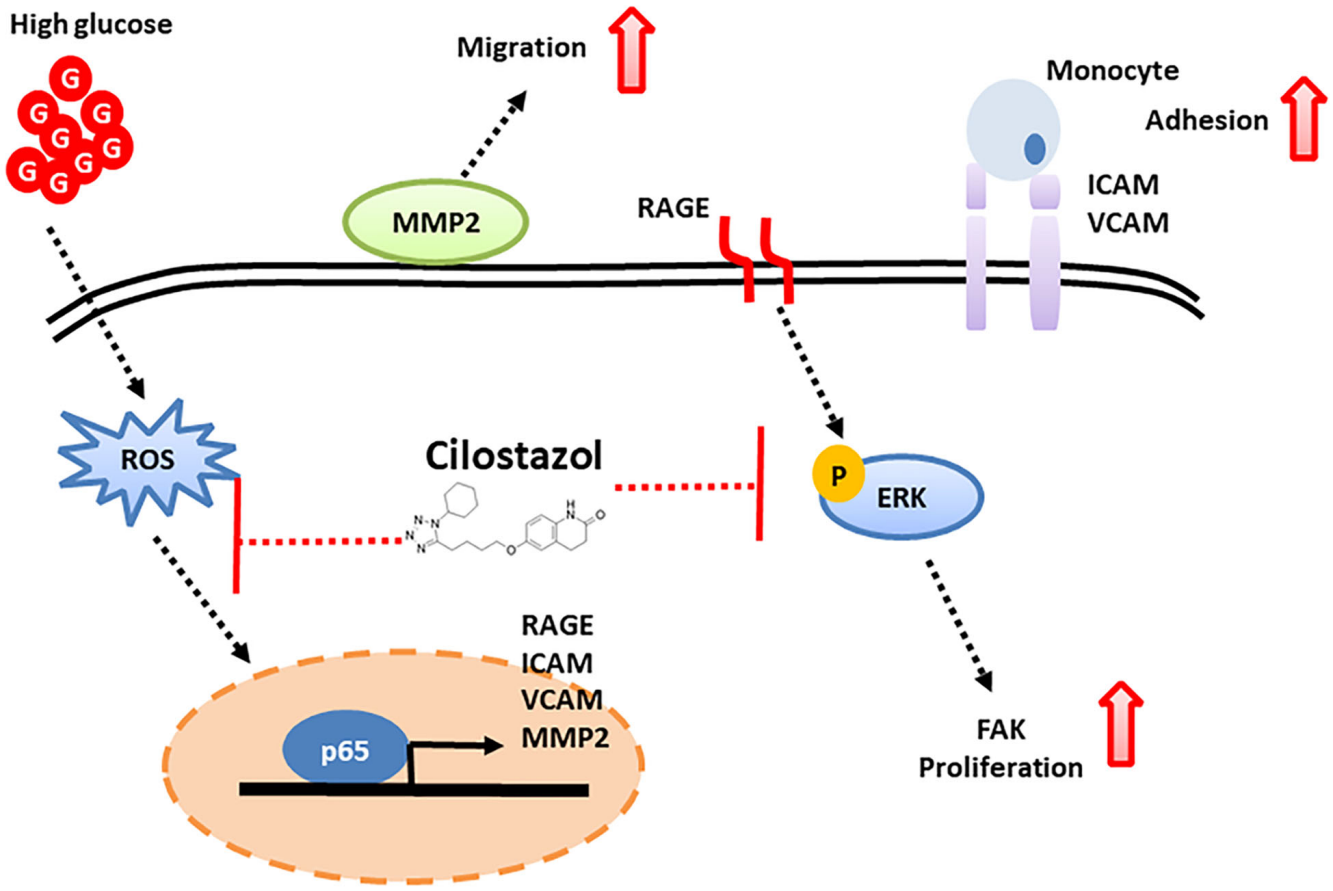
A schematic illustration demonstrated the inhibitory effect of cilostazol on RAGE-mediated oxidative stress, inflammation, adhesion and migration through RAGE/ERK/NF-κB pathways in VSMCs under hyperglycemic conditions

Atherosclerosis is regarded to be a chronic inflammatory disease. In all types of atherosclerosis including that related to diabetes, the accumulation and proliferation of VSMCs in the intima is a key event. VSMC proliferation and migration induced by various growth factors can occur in a variety of pathological processes, including atherosclerosis, hypertension, diabetes, and restenosis after balloon angioplasty [38]. Cilostazol is well known to increase intracellular cAMP levels and to decrease intracellular Ca^2+^ levels, inhibiting platelet aggregation and inducing vasodilatation [39]. A previous study showed that cilostazol could restore HG-induced impairment of endothelial function in endothelial progenitor cells (EPCs) and human umbilical vein endothelial cells (HUVECs) and improve vascular angiogenesis in vitro and in vivo by modulating AMPK/ACC and probably subsequent Akt/eNOS, in parallel with or downstream of the cAMP/PKA-dependent signaling pathway. The authors suggested that cilostazol may provide therapeutic benefits in the treatment of diabetic patients with ischemic disease [40]. Recently, several lines of evidence have suggested that cilostazol has an inhibitory effect on VSMC proliferation. In addition, Aoki et al. demonstrated that cilostazol might attenuate cytokine-induced expressions of the iNOS gene by inhibiting NF-κB following AMPK activation in VSMCs [41]. Kim et al. also reported that cilostazol suppressed VSMC proliferation through the inactivation and downregulation of the transcription factor E2F or AMPK activation induced by HG or platelet-derived growth factor (PDGF)-BB, respectively [12, 42]. Yoo et al. also reported that cilostazol inhibited FBS-stimulated VSMC proliferation through the inhibition of ERK [43]. The authors clearly demonstrated that cilostazol significantly inhibited IL-1-induced ADAM17 expression and ERK phosphorylation in VSMCs, indicating the inhibitory effect of cilostazol on VSMC proliferation. Taken together, the therapeutic effects of cilostazol on HG induced VSMC dysfunction were demonstrated to be mainly through both inhibiting RAGE/ERK/NF-κB pathways and mitigating HG-induced ROS production in our research (Fig. 8), which first indicated that the cilostazol-induced reversal of the HG-induced inhibition of VSMC functions could be mediated through RAGE/ERK/NF-κB signaling pathways, rather than only through anti-oxidative pathways. It has been well known that cilostazol may possess beneficial effects on diabetic nephropathy by means of regulating protein kinase C, TNF-α, TGF-β, and oxidative stress relevant NF-κB activation [36, 44]. Furthermore, cilostazol also appeared to limit cerebral ischemic-reperfusion injury with AGEs by inhibiting transforming growth factor-β1 signaling [45]. Nevertheless, to our knowledge, it was first confirmed in our study that both cilostazol per se and its beneficial effects on downregulation of RAGE expression decreased the HG phosphorylation of ERK in VSMCs. It was also obviously demonstrated that an ERK inhibitor (U0126) significantly eliminated the expressions of HG-induced NF-κB and VSMC functional markers. In addition, cilostazol with U0126 had additive effects on HG-induced VSMC dysfunction, suggesting that ERK is a critical molecule involved in HG-induced VSMC dysfunction. ERK has been reported to be an important molecule for NF-kB activation in VSMCs [46]. We also showed that the NF-kB p65 inhibitor, PDTA, could significantly inhibit HG-induced RAGE, FAK, MMP2, ICAM-1, and VCAM-1 expressions. In addition, additive effects on HG-induced VSMC dysfunction were observed with cilostazol and PDTA treatment. These data demonstrated that cilostazol reversed HG-induced VSMC dysfunction including cell proliferation through activation of RAGE/ERK/NF-kB pathways, which was quite different from that of other research groups 36, 44, 45].

## Conclusions

Given that it remains unclear how cilostazol mitigates diabetes-associated vasculopathy through RAGE and its downstream signaling, the results of this study offer new insight into the signal transduction pathways that regulate cilostazol-mediated protection against HG-induced VSMC injury, and emphasize the involvement of RAGE signaling and downstream RAGE/ERK/NF-κB pathways. The present study also reinforces our clinical published result suggesting that cilostazol may effectively attenuate the severity of PAOD in patients with type 2 diabetes, and that plasma sRAGE is an independent determinant for improving the index of peripheral arterial insufficiency. Identifying signaling intermediaries and transcriptional mediators involved in HG-induced VSMC dysfunction provides an important insight into the pathogenesis of atherosclerosis in patients with diabetes.

## Data Availability

The obtained results of the research are available on reasonable request.

## Abbreviations

ABI: Ankle-brachial index
AGE: Advanced glycation end-product
cAMP: 3′-5′ Cyclic adenosine monophosphate
EPCs: Endothelial progenitor cells
HDL: High-density lipoprotein
HF: High-fat
HG: High glucose
HUVECs: Human umbilical vein endothelial cells
LDL: Low-density lipoprotein
MCP-1: Monocyte chemotactic protein-1
NAC: N-acetylcysteine
NO: Nitric oxide
PAOD: Peripheral arterial occlusive disease
PDE3: Phosphodiesterase type 3
PDGF: Platelet-derived growth factor
RAGE: Receptor for advanced glycation end-products
sRAGE: Soluble form of RAGE
STZ: Streptozotocin
VSMC: Vascular smooth muscle cell
